# Barriers impeding research data sharing on chronic disease prevention among the older adults in low-and middle-income countries: a systematic review

**DOI:** 10.3389/fpubh.2024.1437543

**Published:** 2024-11-29

**Authors:** Neema Florence Vincent Mosha, Patrick Ngulube

**Affiliations:** School of Interdisciplinary Research and Graduate Studies, College of Graduate Studies, University of South Africa, Pretoria, South Africa

**Keywords:** barriers, data sharing, data analytics, older adults, older persons, chronic diseases, prevention

## Abstract

**Introduction:**

Chronic diseases, including cardiovascular disease, diabetes, cancer, and chronic respiratory diseases, are a growing public health concern in low-and middle-income countries (LMICs) among the older population. The current review aimed to identify the main barriers that impede researchers from sharing research data on the prevention of chronic diseases in older adults living in LMICs). The review included both older women and men from these countries.

**Methods:**

Studies were selected from 11 databases, including Web of Science, Scopus, PubMed, Taylor and Francis, Biomedical Central, BioOne, CINAHL, EBSCOHost, ScienceDirect, Wiley Online, and Google Scholar, were then transferred to CADIMA, an online tool for screening purposes, and a total of 1,305,316 studies were identified through a robust search strategy. CADIMA also ensured the quality of all studies in this review. The sampling techniques were performed by selecting and screening studies per this review’s eligibility criteria. Ultimately, 13 studies were found to meet these criteria. A PRISMA flow chart was used to map out the number of studies that were identified, included, and excluded.

**Results:**

Five main barriers were consistently highlighted, including a lack of necessary resources (9, 69%), dealing with complex and sensitive research data (2,15%), lack of policies, procedures, guidelines (5,38%), medical big data processing and integration (2,15%), and inadequate ethical considerations, legal compliance, and privacy protection (6,46%). Discussion: By shedding light on these obstacles, researchers can develop strategies to overcome the identified barriers and address areas requiring further investigation. The registration details of this review can be found under PROSPERO 2023 CRD42023437385, underscoring the importance of this review in advancing our collective understanding of chronic disease prevention among older adults worldwide.

**Systematic review registration:**

PROSPERO, identifier CRD42023437385, available at: https://www.crd.york.ac.uk/prospero/display_record.php?ID=CRD42023437385.

## Introduction

### Background information

Research data sharing has become crucial for enhancing open communication, accessibility, and the reuse of data collected by researchers, ultimately benefiting society. Ramstrand et al. (1) emphasized that sharing research data enhances the quality and transparency of research, encouraging researchers to disseminate the research data generated from their studies. Efforts to promote research data sharing, particularly for chronic disease prevention, are gaining traction, especially in low-and middle-income countries (LMICs) (2, 3). A survey conducted across 13 subSaharan African nations indicated that scientists recognize that sharing research data increases the impact and visibility of their work (4). Established the African Open Science Platform Initiative (AOSP) in 2016, aimed at advancing open science and research collaboration in Africa, reflecting a strong commitment to research data-sharing principles (5). Rooted in the open access movement, research data sharing emphasises the need for improved access, which can lead to significant advancements in human welfare through enhanced analytical methods (6, 7). Research indicates that many researchers acknowledge the benefits of research data sharing and reuse, with the majority having shared their research data at least once (8). Both researchers and policymakers advocate for increased transparency and public accessibility of research data sharing (8, 9).

Furthermore, sharing research data can reduce costs by avoiding duplication and promoting efficiency, even on limited budgets (10, 11), and it can provide more knowledge and insights for new researchers (10). The methodologies employed in big data have significantly advanced over the past decade, often utilizing hypothesis-free approaches such as data mining (12). Although many experts urge researchers to embrace big data techniques, the adoption has been relatively slow (13). While various strategies enhance access to research data, hosting it in open research data repositories (RDRs) is increasingly recommended by publishers, institutions, and funding agencies (14). This approach improves data availability, facilitates ease of search, allows authors to select licenses governing data access and usage, and assigns digital object identifiers (DOIs) for perpetual citation (1). Several repositories compile and evaluate evidence for specific preventive interventions, helping potential adopters cater to community needs, such as the Centres for Diseases Control and Prevention (CDC) Best Practices Clearinghouse for Heart Disease and Stroke Prevention and Management (15). Research data sharing impacts various fields, including health and medical imaging (15). In this context, medical researchers recognize the importance of sharing research data to control and prevent diseases (16). Despite the sensitivities and restrictions surrounding secondary use, Cascini et al. (17) also recognized the necessity of sharing medical research data for disease control and prevention to inform the public on how they can be prevented.

### Research data sharing for disease prevention

In recent years, emerging technologies such as Artificial Intelligence (AI), the Internet of Things (IoT), Big Data Analytics (BDA), cloud storage, telemedicine, computer-aided decision-making, and precision medicine have emerged as promising solutions to contemporary medical challenges (18). These technologies enable researchers to collect, store, and share data more efficiently and facilitate more ways to access these data for public use (12). The movement toward research data sharing gained significant momentum during the COVID-19 pandemic, with the World Health Organization (WHO) emphasizing its critical role in managing the outbreak (19). Organizations like the Global Alliance for Genomics and Health (GA4GH) and numerous global research institutions have recognized that research data sharing fundamentally enhances understanding of diseases, subsequently leading to improvements in diagnosis, treatment, potential cures, and prevention (20, 21). Again, the National Institutes of Health (NIH) in the United States highlights the importance of sharing patient-derived research data to benefit communities at large (22). These initiatives align with the open data movement, which advocates for the broad accessibility of research data with minimal restrictions (23–25).

Literature indicates that research data sharing enhances clinical studies’ quality and transparency (1). Integrating data science and AI further propels advancements in understanding diseases and improving healthcare outcomes (10, 26). The collaboration between AI and big data continuously yields innovative algorithms for disease prediction, diagnosis, and forecasting therapeutic outcomes (27). To fully harness the benefits of data sharing, fostering a culture of responsible sharing within medical and patient communities is essential (26). While ethical considerations support effective data sharing in global health research, it is crucial to proceed cautiously to avoid potential negative impacts on vulnerable populations, such as rights violations, loss of trust, or undermining local capacities (14, 28, 29). Among the diseases requiring special attention are chronic diseases, particularly as they predominantly affect the older adults population. Research data sharing plays a vital role in safeguarding the health of older people (30–32).

Chronic diseases, or non-communicable diseases (NCDs), primarily impact those aged 50 and above and can be easily prevented if the population is well-informed (19) and participates fully in various campaigns aiming to prevent these diseases and willingness to share their health information. According to the WHO, approximately 17 million individuals die from NCDs before the age of 70 each year, with 86% of these premature deaths occurring in LMICs. Annually, chronic diseases account for 41 million deaths, representing 74% of all global mortality (19). These conditions are typically long-lasting (lasting three months or more) and can often be managed but not always cured (33, 34). The classification of chronic diseases varies, with the Centers for Disease Control and Prevention (CDC) identifying conditions such as heart disease, stroke, cancer, type 2 diabetes, obesity, and arthritis as chronic. In contrast, the Centers for Medicare and Medicaid Services (CMS) recognizes a broader list of 19 chronic diseases, including Alzheimer’s disease, depression, and HIV (35).

Aging is associated with increased adiposity and decreased muscle mass, heightening the risk of various chronic diseases, including diabetes, stroke, and cardiovascular conditions (36, 37). Key risk factors for chronic diseases such as tobacco use, physical inactivity, excessive alcohol consumption, poor diet, and air pollution underscore the need for effective prevention strategies (19). Prevention is particularly crucial for populations facing higher chronic disease risks due to poverty or racial/ethnic discrimination, which can hinder access to essential social and economic determinants of health, including healthcare, housing, transportation, and employment (38). Despite the existence of chronic disease prevention measures, such as Human Papillomavirus (HPV) vaccinations and early cancer detection, there remains untapped potential for implementing and sharing research data effectively (38). Researchers collect data from various trials and clinical studies to develop new therapies and improve patient care through evidence-based practices (2). The overarching goal is to alleviate the disproportionate burden of chronic diseases in vulnerable populations (20). Thus, the importance of sharing research data on preventing chronic diseases among the older adults cannot be overstated (39, 40).

### Research question

The research question addressed in this review was “What are the barriers to sharing research data on chronic disease prevention among the older adults in LMICs, and how do these barriers affect health outcomes for this population?”

### Contribution of the review

The main contribution of this article lies in its comprehensive examination of the barriers that impede the sharing of research data related to chronic disease prevention among older persons. While existing literature has identified several challenges, this work highlights lesser-known barriers, such as cultural attitudes toward research data sharing and the impact of digital literacy among older people. The review incorporates insights from diverse stakeholders, including researchers, healthcare providers, and older patients, providing a multifaceted view of the issue. This review identifies barriers and suggests actionable strategies to enhance research data sharing, such as targeted training programs and improved communication channels. These strategies are integrated under barriers. It discusses the implications for policy changes that could facilitate research data sharing, thereby encouraging collaboration across sectors. Thus, by extending the conversation beyond well-documented barriers, this work contributes to a deeper understanding of the complexities involved in data sharing for chronic disease prevention, ultimately aiming to foster more effective interventions for older people.

### Significance of the review

This systematic review investigates the barriers impeding research data sharing on chronic disease prevention among the older adults in LMICs. A comprehensive search was conducted across multiple databases, using specific keywords related to chronic diseases, older people, and research data-sharing practices. The methodological framework employed the Cochrane Systematic Review Guidelines and The Preferred Items for Systematic Reviews and Meta-Analyses (PRISMA) checklist (41) guidelines to ensure rigorous selection and analysis of relevant studies which addresses the barriers researchers face in sharing research data for the benefit of the public. The significance of this study lies in its potential to inform policymakers, decision-makers, and researchers about the challenges researchers face in research data sharing, ultimately facilitating improved collaboration and resource allocation for chronic disease prevention initiatives in LMICs. By addressing these barriers, the findings aim to enhance the value and accessibility of health research data, leading to better health outcomes for the older adults.

### Statement problem

Sharing research data for chronic disease prevention presents several challenges for researchers and patients alike (15). One significant concern is the fear among researchers that their data may be misused or misinterpreted (42). Additionally, there are worries about protecting and controlling research data when entrusted to third parties, such as repository owners (4, 14, 43). Before data sharing can occur, researchers must also secure the willingness of patients to let their data be shared publicly, which is also a critical challenge when they refuse to comply (17). Furthermore, research data sharing challenges traditional notions of intellectual ownership (44). Obtaining patient consent to share research data involves navigating complex consent protocols (22). Issues related to individual privacy, governance of international data transfers, and ethical considerations concerning using personal data for public health purposes are also critical (45). Competitive research practices often discourage research data sharing, as researchers may withhold datasets to protect their career interests and maintain the ability to publish innovative, high-impact studies (46). Concerns about intellectual property (IP) and commercial applications create additional barriers to the secondary use of health data beyond its original purpose (46). Consequently, researchers in LMICs face unique challenges that impede their involvement in the open science movement. Specifically, LMIC researchers often lack access to essential resources, technical support, and adequate infrastructure (4). Common issues include slow or uninterrupted internet connectivity, outdated software and hardware, and frequent power interruptions (47). Discussions surrounding data sharing have predominantly occurred in developed countries, focusing on differences in research data types rather than the specific challenges faced in varied research contexts, particularly in LMICs (48). To encourage greater adoption of open science and data sharing in LMICs, it is vital to understand the barriers hindering the sharing of research data for chronic disease control among older people in these regions. Therefore, this review identified and discussed various barriers that hinder researchers from sharing research data for chronic disease prevention among older persons. By identifying these barriers will give policymakers and decision-makers a clearer understanding necessary to develop effective, long-term solutions.

## Methodology

### Eligibility criteria

The review included all peer-reviewed articles published in the English Language and published between 2013 and 2023. Eligible studies for inclusion had to meet the following criteria: (a) any study published in a peer-reviewed journal; (b) survey, qualitative studies (documentary, semistructured interviews, observations, and case studies), mixed methods research studies; (c) Research articles that explore barriers to sharing research data sharing on chronic diseases in LMICs and presents conclusions and discussions that offer transferable insights to LMICs; and (d) Articles that were not retracted. The exclusion criteria were: (a) Case–control, randomized control trials studies; (b) Peer-reviewed reviews (systematic, scoping, rapid), meta-analysis, reports, books, book chapters, conference proceedings, dissertations and theses, and unpublished manuscripts; (c) Research articles that do not specifically address barriers to sharing research data for chronic diseases LMICs, but whose conclusions and discussions provide transferable insights applicable to LMICs; and (d) Retracted articles.

### Search methods for identification of studies

This study was analyzed and reported using the PRISMA (41). We searched Web of Science, Scopus, PubMed, Taylor and Francis, Biomedical Central, BioOne, CINAHL, EBSCOHost, ScienceDirect, Wiley Online, and Google Scholar for studies published in English using a predefined set of keywords. The researchers created a tailored set of search terms to align with the specific research questions of this review. Boolean operators were employed to optimize the search effectiveness within the chosen databases. Keywords were used to retrieve relevant literature from the selected databases. The retrieved articles were imported into the Zotero reference manager for screening, and the final list was then transferred to CADIMA, a free web tool designed to facilitate the documentation and conduct of systematic reviews, systematic maps, and other literature reviews (49). This tool aims to enhance the efficiency of evidence synthesis and ensure comprehensive reporting of all activities, thereby optimizing methodological rigor (49). Authors (N.M. and P.N.) independently evaluated the titles and abstracts of the retrieved studies to assess their relevance. Any conflicts were discussed and resolved collaboratively. After excluding articles deemed irrelevant at this initial stage, full-text versions of the remaining articles were obtained and assessed for eligibility, following the same screening process. Data coding was conducted manually using Microsoft Excel^©^. Like other systematic reviews, the coding process focused on the authors, study design, publication date, and findings (50).

A thorough and sensitive search strategy was employed. To compile the final list of electronic databases, a combination of professional experience, library subject guides, and databases focused on research data sharing, chronic disease prevention, the older adults, and barriers to sharing research data was utilized. Following the Campbell Collaboration recommendations, field-specific and multidisciplinary databases were searched (51). The search strategy aimed to retrieve published research articles from peer-reviewed journals. Access to these databases was provided through the University of South Africa (UNISA) in Pretoria, South Africa, and via the Internet when applicable. As mentioned, updating the searches will be essential for identifying newly published research articles that can contribute to this work. Search terms were identified and organized into groups by reviewing keywords and subject headings from a sample of randomly selected relevant articles, as well as subject terms used in databases such as Web of Science, Scopus, PubMed, Taylor and Francis, BioOne, CINAHL, EBSCOHost, ScienceDirect, Wiley Online, and Google Scholar. Given the variability among databases, the final search terms were tailored for each one (see Supplementary Tables S1–S7) by consulting the database thesaurus and piloting the search strings.

When searching Google Scholar, a potential source of bias may arise from the platform’s algorithms, which track user data to generate personalized search results. To mitigate algorithmic bias, systematic reviewers can utilize ‘secure’ search engines that do not track users, such as DuckDuckGo. However, Landerdahl Stridsberg (52) suggested using ‘incognito mode’ in Google or Google Scholar to achieve similar results without bias. For this review, Google Scholar was utilized with search history, location services, and disabled personalization options to prevent tailored results from influencing the findings. The Google Scholar search function is limited to 256 characters (including operators), so a more focused search string was crafted, screening the first 150 hits for relevance. Special attention was given to synonyms, country-specific spellings, and various mitigation and adaptation strategies employed by different farmers.

Using Boolean operators, a sample search string was developed:

- Research data sharing OR “Information sharing” OR “Data sharing” OR “Data exchange” OR “Exchange of data” OR “Data transfer” OR “Open data.”
- AND (Barrier* OR “Challenge*” OR “Problem*” OR “Obstacle*”).
- AND (Older adult OR “Aged” OR “Older”).
- AND (Low-and middle-income country* OR “Economically developing countries*” OR “Developing countries*” OR “Third World Countries” OR “Developing Economies”).

Reviewers conducted independent evaluations based on predefined inclusion and exclusion criteria. Two postdoctoral fellows were invited to evaluate the articles after the initial assessments. Any discrepancies were resolved through discussions between the two reviewers until a consensus was reached. The selected research articles were then re-evaluated, and the agreed-upon articles were analyzed.

### Data collection and analysis

#### Selection of studies

**Figure 1:**
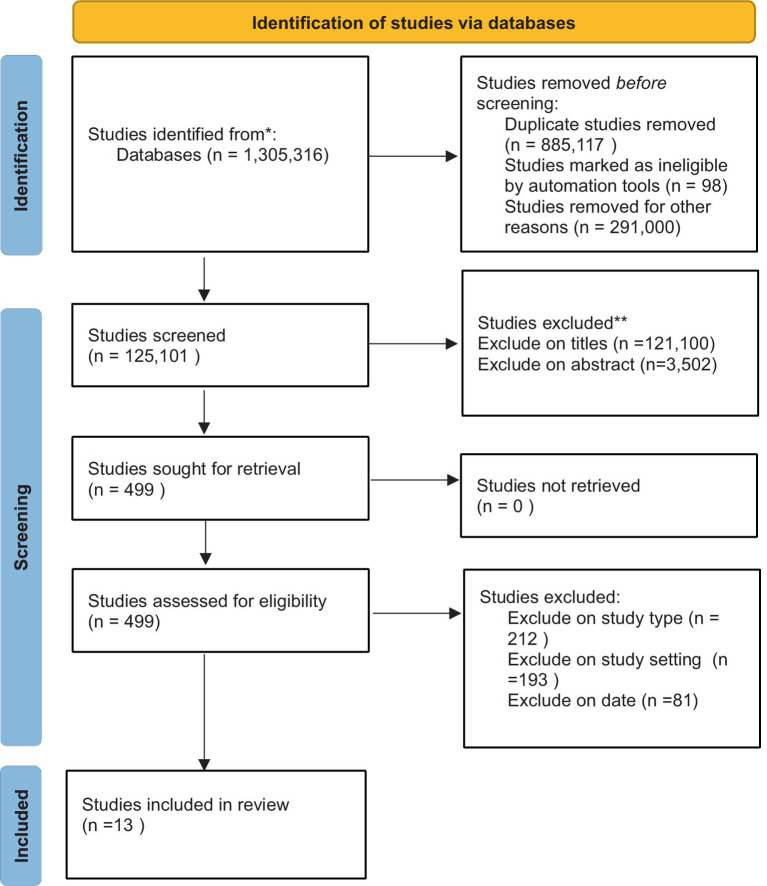
PRISMA flow diagram.

All searches were conducted by the first author (N.M.), with support from the co-author during the screening process. Eligible studies were imported into the open-access online tool CADIMA, developed through a collaboration between the Julius Kühn Institute and the Collaboration for Environmental Evidence (49). This tool aims to enhance the efficiency of the evidence synthesis process and facilitate comprehensive reporting of all activities to maximize methodological rigor (49). Duplicate records were removed, and all available information was gathered from reports of the same study. In the first screening round, the titles and abstracts of the studies were reviewed against the inclusion criteria. The first author screened all remaining records and distributed them to the co-author to ensure independent evaluation of each record. Full texts were retrieved for studies deemed relevant or where their relevance was uncertain, and the dual screening process was repeated to confirm their eligibility. As noted, the first author screened all records while the co-author independently reviewed each selection. Generally, there was fair to moderate or significant agreement between the screeners’ decisions. Any disagreements at each stage of the process were discussed until a consensus was reached. The entire screening process was documented using a PRISMA Flow Diagram, as outlined in Chapter 4 of the Cochrane Handbook (53). Figure 1 presents the flow chart illustrating the search outcomes and the selection process for the research articles included in this review (41).

### Data extraction and management

A data extraction framework, guided by the Template for Intervention Description and Replication (TIDieR) (54), was developed and refined following the identification and review of the final studies. Data extraction focused on key information about the author information, year of publication, study design, study setting, and barriers (Table 1). Again, the first author extracted general review information, while both reviewers independently extracted outcome data.

**Table 1:** Characteristics of the reviewed articles.

Sn	Author(s)	Study design	Study area	Barriers
1	Al-Ebbini et al. (55)	Survey	Jordan	Lack of guidelines, restrictive policies, lack of resources, protection of privacy, ownership issues, lack of motivations, and restrictive data formatting, lack of regulations, belief that data should not be available to the public, no place to deposit the data (data repositories) and/or lack of funding to cover costs of data deposition.
2	Bezuidenhout Chakauya (4)	A quantitative survey	Angola, Botswana, Malawi, Mauritius, Mozambique, Namibia, Lesotho, Swaziland, Seychelles, Madagascar, South Africa, Zambia, and Zimbabwe	The loss of intellectual property rights, being scooped and misinterpretation or misuse of data. ICT limitations in LMICs, such as low processing power for data analysis, curtailed access to online environments, and challenges of lowered connectivity and funds. Lack of online visibility. Particularly, the absence of pre-publication data and the slow rate of academic publications
3	Cheah et al. (56)	Qualitative study with 15 interviews and three focus groups discussions	Thailand	Potential risks associated with sharing sensitive data include concerns about patient confidentiality, insufficient time, funding, and resources, as well as the absence of policies that support research data sharing. Participants may worry that non-medical data, such as ethnicity, GPS locations, and patient addresses, could also be sensitive in certain contexts. Researchers have reported feeling inadequately acknowledged by those who utilize the data they have generated. Additionally, there is a lack of agreed-upon consent models to encourage participants’ willingness to share their data.
4	Denny et al. (39)	Qualitative study involving stakeholders	South Africa	Lack of open data sharing policy. Financial constraints was also observed. Informed consent from participants to express their consent for the sharing of their data. Right and ownership of data
5	Kaewkungwal et al. (43)	Survey with researchers worked in biomedical and related research	Thailand	Researchers were less concerned with informed consent and the feasibility of conducting research and sharing data. Ethical and legal compliance in sharing individual data, control of the use of “sensitive” or “restricted” data by other researchers, and proprietary data. Lack of necessary resources (time and money)
6	Jao et al. (63)	Qualitative study involving stakeholders	Kenya	Lack of national research governance policy guiding public health research data sharing. Less research-experienced stakeholders generally had little awareness that researchers might share data within the scientific community, how this might occur, and what uses this might have for progress in science. Risks of loss of privacy and stigmatization among participants
7	Parker and Bull (57)	Qualitative studies (face to face meeting)	India, Kenya, South Africa, Thailand, and Vietnam.	There is a lack of effective data-sharing models that can inspire trust and confidence among relevant stakeholders. Sharing research data from genomic research presents practical ethical and governance challenges.
8	Atakro et al. (64)	Qualitative exploratory descriptive study design	Ghana	There is an absence of policies governing the exchange and sharing of data and information related to individuals’ health conditions.
9	Lin et al. (58)	Survey investigate recent research efforts, and conducts a comprehensive overview of the work on medical big data.	China	Lack of modelling methods and software techniques for integrating complex healthcare data with big data collected from patients, as well as a deficiency in medical multi-data source integration. Lack of knowledge and capacity of handling big data generated from patients with chronic diseases. Big data analytics awareness among researchers. Key elements such as research data preprocessing, data modelling, data visualization, and security are missing from the data management and analysis processes.
10	Zhang (59)	Survey	China	Mishandling of the large volumes of research data generated upon patient admission to hospitals, including laboratory results, medication records, fluid balance, progress notes, and medical imaging. Insufficient effort to fully leverage the big data continuously generated by electronic medical record (EMR) systems and other healthcare databases. A lack of advanced analytics techniques for utilizing big data, particularly in hospitals located in major cities like those in China. Deficiency in technologies designed to utilize the big data produced by EMR systems and other healthcare databases.
11	Belachew et al. (60)	Cross-sectional	Ethiopia	Patients often show reluctance to share their data. Shortage of resources, such as internet access and mobile phones, that hinders data sharing among patients.
12	Naz et al. (61)	Survey	Pakistan	Insufficient evidence guiding health decisions, leading to a lack of confidence among policymakers and decision-makers regarding the use of research data collected from patients. Lack of open and non-judgmental discussions about data quality during the stages of acquisition, transmission, storage, and throughout various analyses and reporting processes.
13	Kim and Choi (62)		China	Older adults often have a mistrust of government agencies when it comes to sharing their personal information. There is a significant gap in healthcare technology and services available to older adults, particularly within public health systems. Privacy concerns and diversity issues are particularly pronounced among older adults, especially among older women.

## Results

Thirteen (13) research articles were analyzed, and the results are presented below:

### Countries included in this review

Of the 13 studies included, there was one from Jordan, one from South Africa, two from Thailand, one from Kenya, one from Ghana, one from Pakistan, one from Ethiopia, and three from China. Additionally, two studies encompassed multiple LMICs; one included Angola, Botswana, Malawi, Mauritius, Mozambique, Namibia, Lesotho, Swaziland, Seychelles, Madagascar, South Africa, Zambia, and Zimbabwe, while the other covered India, Kenya, South Africa, Thailand, and Vietnam.

### Year of publication

While the search criteria encompassed studies from 2014 to 2023 (Figure 1). Most of the reviewed articles 4 (31%) were published in 2015. This demonstrates a small but growing interest in this area of research data management and sharing and the need for further research. There are also several studies by the same research area that may account for this spike in studies. An update to this systematic review in future years will be important to clarify if there has indeed been a rise in research interest.

### Necessary resources (time, funds, and infrastructure)

Eleven reviewed articles (*n* = 11; 85%) identified a lack of necessary resources such as time, funds, infrastructure and model methods, and software techniques to facilitate the sharing of research data for preventing chronic diseases among older people in LMICs (4, 39, 43, 55–62). Al-Ebbini et al. (55) highlighted the insufficiency of resources like open RDRs and insufficient funding to cover the expenses of depositing research data into paid RDRs and public sharing of research. Kaewkungwal et al. (43) noted that only around 20% of respondents in LMICs considered the availability of necessary resources (time, money, and infrastructure) to be a significant challenge. Belachew et al. (60) highlighted the lack of resources for research data sharing, including the Internet and cell phones.

Bezuidenhout and Chakauya (4) highlighted various challenges LMICs face, such as limited processing power for data analysis, restricted access to online platforms, connectivity, and financial constraints. Lin et al. (58) highlighted the lack of health-and healthcare-related content generated from numerous patient care points of contact, sophisticated medical instruments, and web-based health communities; the lack of modelling methods and software techniques for blending complex healthcare data, advanced analytic tools, and lack of distributed scientific computing, and incapability of handling the huge amount of complex data with high volume, high velocity, and high variety. Kim and Choi (62) highlighted poor healthcare technology and relevant services, particularly in public health. These challenges limit online visibility, particularly the absence of pre-publication research data and the slow pace of academic publications (4). This diminishes the global impact of research from LMICs. A lack of funding for supporting researchers in research data analysis, sharing, and providing feedback to participants was presented by Denny et al. (39). Moreover, Bezuidenhout and Chakauya (4) emphasized that the slow publication rate leads researchers in LMICs to be hesitant about sharing pre-publication data with other researchers outside their personal network. They also highlighted the reluctance of LMIC researchers to engage in alternative pathways, indicating a need to explore the concept of “personal connections” further for these researchers. Cheah et al. (56) suggested that having a gatekeeper or committee to support research activities, including research data sharing, could result in additional expenses, necessitating a clear identification of costs and required personnel in the project budgets.

Denny et al. (39) proposed that participant input and expenses related to research data curation should be included as standard budget allocations in all research grants and mandated as a prerequisite for ethical approval. Zhang et al. (59) highlighted a deficiency in technologies that utilize the big data produced by EMR systems and other healthcare databases. Naz et al. (61) highlighted the lack of technologies to enhance data quality at acquisition, transmission, and storage and various analysis and reporting equipment. Allocating sufficient funds, time, and resources beyond the original study duration is crucial since research data sharing may extend well beyond the conclusion of the initial research project, potentially discouraging applicants and research data users unfamiliar with the specific access requirements (56). This challenge could disproportionately affect individuals from LMICs (4). Cheah et al. (56) also reported on the lack of researchers’ experiences and confidence in sharing research data, with most researchers having limited personal research data sharing experiences.

### Complex and sensitive research data

Two reviewed research articles (*n* = 2, 15%) explored the complexities of research data containing sensitive and sensitive information that could potentially harm subjects or expose them to legal action or reputational damage (43, 56). Kaewkungwal et al. (43) identified sensitive research data, such as details on sexual behavior and mental health conditions related to patients’ diseases and conditions, as particularly vulnerable to misuse of research data and statistics about older persons (43). Cheah et al. (56) discussed a lack of established consent models and effective methods to empower research participants in making decisions about using their research data. While some individuals supported obtaining consent for data utilization, others found the process burdensome, as it can be difficult to explain the wide range of potential research data uses and the various entities that may seek access to their information (56). Despite recognizing the benefits of research data sharing in enhancing scientific progress, improving data analysis, and optimizing resource allocation, concerns remained regarding the risks posed to participants and the research itself (56). Cheah et al. (56) also emphasized the importance of addressing these risks to promote research data sharing within the scientific community. Participants offered recommendations on mitigating potential dangers associated with research data sharing, aiming to facilitate the realisation of research data sharing while safeguarding the interests of all involved parties (56).

### Policies, procedures, and guidelines

Five research articles (*n* = 5; 38%) demonstrated that promoting research data sharing for chronic diseases in LMICs requires the establishment of robust policies and procedures (39, 56, 61, 63, 64). Cheah et al. (56) stressed the importance of evaluating existing policies, procedures, and guidelines to promote research data sharing and assess their relevance and efficacy within LMIC settings. Reviewed articles indicated a notable absence of research data-sharing policies and operational procedures across various disease prevention in LMICs (56, 63). Additionally, a lack of open data-sharing policies was identified as a barrier to enhancing research data-sharing (39). A reviewed article by Denny et al. (39) further discussed the challenges associated with shifting organisational attitudes and fostering a culture of research data sharing when introducing new open policies. The reviewed articles revealed a dearth of evidence concerning the analysis and implementation of research data-sharing policies, monitoring of policy compliance, clarity in establishing data-sharing rules and procedures, and enforcing discipline standards (56). Atakro et al. (64) highlighted the lack of policies regarding the exchange and sharing of research data and information regarding individuals’ health conditions. Naz et al. (61) highlighted the lack of evidence in health decisions and the policy and decision-makers’ confidence in using research data collected from patients. Cheah et al. (56) reiterated the critical need for research data-sharing policies and procedures in LMICs to promote equitable access to research data, emphasizing the importance of establishing sustainable capabilities for sharing and analyzing raw data and datasets. The insights gleaned from the reviewed paper by Cheah et al. (56) were instrumental in formulating policies and procedures for research data sharing, focusing on minimizing potential risks while upholding the trust and confidence of researchers, communities, and research participants. Denny et al. (39) held significant policy implications and, in conjunction with insights from collaborations in various countries, including Thailand, India, Vietnam, and Kenya, has the potential to influence future research data-sharing policies in South Africa and other LMICs.

### Medical big data processing and integration

A total of two reviewed research articles (*n* = 2, 15%) highlighted the challenge of handling big data collected from patients with chronic diseases (58, 59). The health community faces challenges of health and healthcare-related content generated from numerous patient care points of contact, sophisticated medical instruments, and web-based health communities (58). Healthcare big data comprises data from different structured, semi-structured, and unstructured sources (58). Zhang et al. (59) highlighted the mishandling of these large volumes of research data generated upon patient admission to hospitals, including laboratory results, medication records, fluid balance, progress notes, and medical imaging. Lin et al. (58) recommended that even though big data holds the promise of supporting a wide range of medical and healthcare functions like disease surveillance, clinical decision support, and population health management, there is a lack of advanced analytic tools and distributed scientific computing for big data processing and integration. Lin et al. (58) also highlighted that a roadmap for uniform handling and analysing such complex data remains elusive without a robust fundamental theory for representation, analysis, and inference. Zhang et al. (59) also added that most hospitals located in big cities cannot handle the huge amount of complex data with high volume, velocity, and variety. For veracity, medical data might be incomplete, biased, or even filled with noise (59). Zhang et al. (59) also added that insufficient effort exists to fully leverage the continuously generated big data by EMR systems and other healthcare databases.

### Ethical considerations, legal compliance, and privacy protection

Lack of ethical considerations, legal compliance, and privacy protection was highlighted by (*n* = 6, 46%) reviewed papers (39, 43, 56, 57, 60, 62). Most of the reviewed articles highlighted the absence of ethical considerations, legal compliance, and privacy protection to enhance research data sharing (39, 57, 62). Researchers argued that sharing valuable research data can have ethical justifications, especially when repeating data collection would be impractical or unethical (56, 57, 62). Cheah et al. (56) highlighted that some researchers provided ethical justifications for research data sharing under specific circumstances, such as when raw data and datasets are highly valuable or in emergency situations. Parker and Bull (57) reported a lack of ethical consideration in most of the research conducted in LMICs that limits the sharing of research data. Reviewed articles also highlighted the lack of legal compliance, ethical clearance, and data security risks that act as obstacles to sharing research data (43, 57, 62). They argued that researchers should ensure they obtain ethical clearance to enable them to share their research data for the benefit of other researchers and institutions. For instance, Denny et al. (39) obtained ethical approval from the Research Ethics Committee (REC) Humanities and Social Science (HSSREC) at the University of KwaZulu-Natal and Oxford Tropical RECs. These ethical approvals were crucial for safeguarding research participants’ rights and well-being (39). Kim and Choi (62) insisted on privacy consideration and diversity among older adults, especially older women. There is also a lack of trust between researchers and patients in relation to sharing research data. Willingness from patients to allow their data to be shared was also reported among the challenges that hinder researchers from researching data collected from patients (60).

## Discussion

Five barriers that hinder the sharing of research data for chronic disease prevention were discussed in this review. The review discussed the lack of necessary resources, including funding, infrastructure, and time to support research data sharing for chronic disease prevention among the older adults in LMICs. Musa et al. (65) highlighted that in many LMICs, health research data sharing is constrained by inadequate infrastructure, limited resources, and cultural challenges, resulting in insufficient data analysis, curation, and sharing. Similarly, Anger et al. (66) reported a lack of infrastructure, low awareness, insufficient funding, and inadequate institutional support. For example, some laboratories may hesitate to invest in data sharing due to high costs that do not provide immediate returns (42). Devriendt et al. (67) discuss the absence of datasharing platforms that facilitate the sharing of cohort study data in biomedical research. Schwalbe et al. (104) also address the shortfall of platforms for clinical data sharing, particularly in fields like cardiovascular disease. However, efforts are emerging to improve drug development and clinical practices. The authors noted a lack of support for researchers in using these platforms for data sharing (67–69). Effective platforms should include technical components like data catalogs, access management systems, and virtual research environments to enhance the findability, accessibility, interoperability, and reusability (FAIR) of research data (67, 68). Devriendt et al. (67) highlight that a lack of motivation, such as inadequate compensation for time and effort, affects research data sharing. Additionally, the absence of academic incentives and recognition for researchers has been noted (66, 70, 71), with some arguing that expecting researchers to share data without sufficient incentives is unreasonable (29). Ensuring the necessary resources to enhance research data sharing for chronic disease prevention among older people is essential for promoting effective research data sharing (66, 71).

This review also discussed sharing complex and sensitive research data containing confidential information. Were and Meslin (72) highlighted that complex and sensitive research data cannot be shared without written consent from both participants and researchers. The absence of clear guidelines and transparency complicates the sharing of this data, especially when there is no agreement from the data owner or patient (70). For instance, cancer patients worldwide have been encouraged to permit the sharing of their data to help the public learn and take preventative measures (32, 73). An example is CANSA’s Online Support Resources, an online community where cancer patients contribute their data to advance scientific research, enabling communities to access support when necessary. Additionally, global collaborations in neuroimaging genetics have emerged to aggregate and compare brain data, facilitating the replication of study findings (72–74). While ethical concerns regarding genetics, neuroimaging, and multi-site collaborative research have received attention, there has been limited discussion on the ethical implications arising from the intersection of these fields in global neuroimaging genetics collaborations (74). Handling clinical trials and patient data also raises ethical and legal questions about data deidentification and re-identification risks (42).

Flexibility in research data-sharing policies and guidelines is essential, emphasizing how these data are shared (75, 76). This review also highlights the absence of policies, procedures, and guidelines as among the key barriers to impede research data sharing. The same observation was noted by Nelson’s study (77), which found that despite widespread institutionalisation of open access data sharing policies across research funding and other agencies, researchers have not fully adopted these policies possible, with instances of resistance observed across the natural and social science disciplines. A study by Bull et al. (78) also noted the scarcity of empirically grounded reports on data release policies for biomedical and public health research in LMICs. Mauthner and Parry (24) add that research data-sharing policies and fundamental scientific, ethical, and political frameworks encapsulate and establish a specific comprehension of scientific and data-sharing practices that yield consequences. LMIC settings require research data-sharing policies to facilitate equitable data utilization and develop sustainable capabilities for sharing and analysing raw data and datasets (75, 76). Furthermore, there are variations in the tone and content of funding agency regulations, which can be prescriptive. For example, the UK’s ESRC mandates that grant recipients deposit research data (and enforces financial penalties for non-compliance) unless valid justifications for on-disclosure exist (79). The implementation of data-sharing policies has faced significant delays among researchers and research organisations (77, 80, 81). Subsequently, in 2010, research funders collaborated to assess the impact of research data policies, making the first concentrated effort to harmonize and address concerns raised by researchers LMICs regarding research data sharing (70).

Medical big data processing and integration are critical issues highlighted in this review. Challenges related to big data analytics and a lack of techniques and expertise among researchers in managing and analyzing these data hinder efforts to share information for chronic disease prevention. The review also found that researchers often collect extensive amounts of data from hospitals and patients, which can lead to mishandling. Fang (82) provided a comprehensive overview of the challenges, techniques, and future directions in computational health informatics within the era of big data, offering a structured analysis of both historical and contemporary methods. Large volumes of data, such as physician notes, medical prescriptions, and lab and imaging reports, remain underutilized without effective methods for real-time interactive processing (83). A primary challenge of big data lies in managing vast amounts of information and leveraging it to make data-driven decisions across various healthcare domains. Additionally, significant challenges are related to the storage, analysis, presentation of results, and clinical inference of big data (84). From a clinical perspective, big data analysis aims to enhance patient health outcomes, enable long-term health predictions, and implement appropriate therapeutic interventions. Chawla et al. (85) laid the groundwork for a Big Data-driven approach to personalized healthcare, demonstrating its relevance to patient-centered outcomes, meaningful use, and reducing readmission rates. With advancements in big data analytics, there has been an increasing focus on disease prediction, utilizing automated feature selection from extensive datasets to improve risk classification accuracy (86, 87). Primary data pools are central to the big data revolution in healthcare, particularly concerning chronic diseases and health monitoring. Integrating research data stored in both structured and unstructured formats can significantly enhance organizational value (88). Archenaa and Mary Anita (83) explored using Apache Spark for predictive analytics in healthcare. Wang et al. (89) proposed an efficient flow estimation algorithm for telehealth cloud systems, along with a data coherence protocol for Personal Health Record (PHR)-based distributed systems. Bates et al. (12) identified six applications of big data in healthcare. Qiu et al. (86) presented an optimal big data sharing algorithm to manage complex datasets in telehealth using cloud technologies. Krumholz (90) introduced a cyber-physical system for patient-centric healthcare applications called Health-CPS, which featured a unified data collection layer and a data-oriented service layer. The findings indicated that cloud and big data technologies can significantly enhance healthcare system performance. Oliver et al. (91) described a novel class of recursive query answering plans, further contributing to the discussion on effective data management in healthcare contexts.

Ethical considerations, legal compliance, and privacy protection are additional barriers highlighted in this review paper. This barrier underscores the importance of participants’ consent and confidentiality in determining the willingness to share research data. Ethical factors, including data privacy, security, ownership, and informed consent, significantly limit research data sharing (72). Privacy concerns and the need for participants’ consent are vital in public health (92). Ethical protocols are established to ensure adherence to ethical and legal standards, restricting data reuse to legitimate researchers (93). For successful data-sharing initiatives, it is essential to ensure informed consent and data anonymization and address ethical and legal responsibilities (24). International research data sharing should benefit both individuals and society while respecting the rights of data collectors and producers (26). Addressing legal, ethical, and privacy issues is critical for promoting effective and ethical research data sharing (94, 95). Privacy concerns among providers can hinder the disclosure of patient information for public health purposes, even in cases of mandated disease reporting (92). In pandemic situations, some argue that public interest should precede individual privacy rights (92). Conversely, challenges inherent in research data sharing include ethical considerations in participant recruitment and establishing clear agreements on data linkage and release (96). Trust plays a significant role in individuals’ willingness to share sensitive information, influenced by their familiarity with the person or organization requesting the data (97). Bull et al. (78) suggested four key elements of ethical issues related to research data sharing in low LMICs have been proposed: assessing the value of sharing by engaging all stakeholders, minimizing harm to participants and populations, promoting fairness and reciprocity by protecting local researchers’ interests, and fostering trust among stakeholders. Furthermore, there is a need for additional research on broad consent procedures, governance models, data-sharing policies, and capacity building (98). Additionally, various studies have identified other barriers, such as socio-cultural obstacles and a lack of a culture of data sharing, which complicates introducing new initiatives (99). There is also a deficiency in metadata and metadata standards that describe data content, origin, methods, secondary data use, and interoperability (100–102).

### Related work

Among the related work is the systematic review by Panhuis et al. (103), titled “A Systematic Review of Barriers to Data Sharing in Public Health,” which examines data sharing within the broader context of public health rather than focusing on specific populations. The review identifies several barriers to data sharing, including:

- Technical challenges: These include issues such as inadequate metadata and standards, language barriers, unavailability of technical solutions, unpreserved research data, difficulty in locating data, and poorly collected data.
- Motivational barriers: the authors highlight challenges related to a lack of incentives and motivation among researchers, opportunity costs, potential criticism, and disagreements regarding data usage.
- Economic barriers: these encompass concerns about potential economic repercussions and insufficient resources for data sharing.
- Political barriers: challenges in this area include a lack of trust, restrictive policies, and insufficient guidelines for data sharing.
- Legal and ethical barriers: the review discusses various legal and ethical considerations impeding effective research data-sharing practices.

The current study differs from prior studies already conducted in the study area.

- Focus on specific populations: unlike previous studies that broadly addressed data sharing in public health, our current study specifically targets chronic disease prevention among the older adults. This focused approach allows for a deeper understanding of this demographic’s unique barriers and facilitators.
- Contextual relevance: While earlier reviews may have generalized findings across various public health contexts, our study emphasizes the unique challenges faced in LMICs. By concentrating on this specific setting, we aim to provide more applicable insights into the local healthcare environment.
- Comprehensive barrier analysis: Previous studies often categorized barriers into specific categories to understand in detail how these barriers affect researchers. This nuanced approach facilitates a more thorough exploration of each barrier’s impact on research data sharing.

## Conclusion

This systematic review underscores the significant barriers to research data sharing on chronic disease prevention among older adults in LMICs. Ethical considerations, legal compliance, and privacy protection are paramount, highlighting the necessity for informed consent and confidentiality to foster patient trust. Barriers include a lack of necessary resources, the sharing of complex and sensitive research data, the absence of policies, procedures, and guidelines, and the slow processing and integration of big medical data. While these barriers pose difficulties, many can be mitigated through policy adjustments, especially by funding bodies and journals offering backing and acknowledgment to researchers engaging in data-sharing efforts. It is thus essential to develop clear policies, invest in infrastructure, and promote training programs that facilitate ethical data-sharing practices. Engaging stakeholders, including local researchers and communities, is crucial to ensure that research efforts align with the health needs of populations. It is also imperative to establish a global framework to optimize existing solutions and devise new strategies for enhancing the utilization and sharing of research data to improve public health in the 21st century. Consequently, sharing research data concerning the prevention of chronic diseases like cancer, type 2 diabetes, and stroke involves navigating intricate ethical considerations. Thus, by involving all stakeholders in a collaborative effort and enforcing robust ethical guidelines, we can establish a pathway for the responsible and impactful sharing of research data for disease prevention, benefiting patients, researchers, and society. Future research should focus on establishing governance models, broad consent procedures, and capacity-building initiatives that promote ethical data sharing. By addressing these barriers and emphasizing the importance of collaboration, we can enhance the effectiveness of chronic disease prevention strategies and ultimately improve health outcomes for older persons in these regions.

### Study implications

The findings identified specific barriers to research data sharing in low-and middle-income countries (LMICs), especially regarding chronic disease prevention among the older adults. This review underscores the need for training programs to enhance researchers’ data management and sharing skills, which can lead to more efficient and effective health interventions. It also highlights the importance of prioritizing funding and resources by policymakers and medical organizations to establish the necessary infrastructure for data sharing, ensuring collaborative systems are in place. Additionally, the review outlines guidelines and policies that promote research data-sharing practices while establishing ethical standards and protecting patient privacy, all in the interest of fostering transparency in research. The implications for researchers include identifying barriers that can inform future studies focused on specific challenges in data sharing within LMICs, paving the way for targeted solutions and innovations. Socially, this review advocates for collaboration among researchers from diverse disciplines, which can enrich the research findings, especially in tackling complex health issues like chronic disease prevention. Ultimately, the study supports developing and accessing interventions to enhance research data-sharing practices, offering a framework for evaluating their effects on health outcomes and research effectiveness.

### Limitation and future study

The review may not encompass all LMICs, potentially limiting the findings’ generalizability. It utilised published articles, which may overlook valuable data from unpublished studies or grey literature, leading to bias in the results. Differences in how chronic disease prevention and research data sharing are defined across studies may affect the consistency and comparability of findings. The quality of the studies reviewed varies, which may influence the robustness of the conclusions drawn from the synthesized data. The review included studies published in English and potentially excluded relevant research published in other languages. Lastly, this review identified the barriers, but it did not address all factors influencing research data sharing, such as cultural or social influences that could impact the findings.

### Recommendations

1. Create and implement clear policies that promote and facilitate research data sharing, specifically tailored to the context of chronic disease prevention among older adults.
2. Invest in the necessary technological infrastructure to support secure and efficient research data sharing, including reliable internet access and research data management systems.
3. Develop training programs for researchers and healthcare professionals focusing on data sharing best practices, ethics, and the use of technology in research data management.
4. Facilitate collaborative research initiatives that bring together various stakeholders, including academic institutions, healthcare providers, and community organizations, to share data and resources.
5. Create standardized data formats and protocols to ensure consistency and ease of sharing across different research studies and healthcare systems.
6. Establish guidelines addressing ethical considerations regarding research data sharing, particularly concerning patient privacy and consent.
7. Conduct awareness campaigns to educate stakeholders about the benefits of research data sharing for improving chronic disease prevention and management among older adults.
8. Engage the older persons in research data-sharing discussions to understand their concerns and preferences, ensuring that their perspectives are considered in policy development.

## Data Availability

The original contributions presented in the study are included in the article/Supplementary material, further inquiries can be directed to the corresponding author.
