# Supplementary material for: Barriers impeding research data sharing on chronic disease prevention among the older adults in low-and middle-income countries: a systematic review
Source: Front Public Health. 2024 Nov 29;12:1437543. doi: 10.3389/fpubh.2024.1437543 (PMC11638978; doi:10.3389/fpubh.2024.1437543)
Supplement: Supplementary file 1 [file Table_1.DOCX]

**Supplementary file 1: Search strategy – 04/12/2023**

**Table 1: Web of Science (WoS) = 964**

| Filter | Search statement |
| --- | --- |
| All fields | #1: Research data OR “Research evidence” OR “Research finding*” OR “Survey data” OR “Scientific evidence” OR “Research result*” OR “Scientific data” |
| All fields | #2: Research data sharing OR “Information sharing” OR “Data sharing” OR “Data exchange” OR “Exchange of data” OR “Data transfer” OR “Open data” |
| All fields | #3: Barrier* OR “Challenge*” OR “Problem*” OR “Obstacle*” |
| All fields | #4: Elderly OR “Aged” OR “Older” |
| All fields | #5: Low- and middle-income country* OR “Economically developing countries*” OR “Developing countries*” OR “Third World Countries” OR “Developing Economies” |
|  | #1 AND # 2 AND #3 AND # 4 AND # 5 |

**Table 2: Web of Science (WoS) = 964**

| Filter | Search statement |
| --- | --- |
| All fields | #1: Research data OR “Research evidence” OR “Research finding*” OR “Survey data” OR “Scientific evidence” OR “Research result*” OR “Scientific data” |
| All fields | #2: Research data sharing OR “Information sharing” OR “Data sharing” OR “Data exchange” OR “Exchange of data” OR “Data transfer” OR “Open data” |
| All fields | #3: Barrier* OR “Challenge*” OR “Problem*” OR “Obstacle*” |
| All fields | #4: Elderly OR “Aged” OR “Older” |
| All fields | #5: Low- and middle-income country* OR “Economically developing countries*” OR “Developing countries*” OR “Third World Countries” OR “Developing Economies” |
|  | #1 AND # 2 AND #3 AND # 4 AND # 5 |

**Table 3: Web of Science (WoS) = 964**

| Filter | Search statement |
| --- | --- |
| All fields | #1: Research data OR “Research evidence” OR “Research finding*” OR “Survey data” OR “Scientific evidence” OR “Research result*” OR “Scientific data” |
| All fields | #2: Research data sharing OR “Information sharing” OR “Data sharing” OR “Data exchange” OR “Exchange of data” OR “Data transfer” OR “Open data” |
| All fields | #3: Chronic disease* OR “Non communicable disease*” OR “Cancer” OR “Diabetic” OR “Stroke” OR “Arthritis*” OR “Epilepsy” OR “Obesity” OR “Chronic illness” OR “Depression” OR “cardiovascular disease*” OR “Alzheimer’s disease*” OR “chronic kidney disease*” OR “Osteoporosis” OR “Type 2 diabetes” |
| All fields | #4: Barrier* OR “Challenge*” OR “Problem*” OR “Obstacle*” |
| All fields | #5: Elderly OR “Aged” OR “Older” |
| All fields | #6: Low- and middle-income country* OR “Economically developing countries*” OR “Developing countries*” OR “Third World Countries” OR “Developing Economies” |
|  | #1 AND # 2 AND #3 AND # 4 AND # 5 AND # 6 |

**Table 4: Scopus = 821**

| Filter | Search statement |
| --- | --- |
| Title/Abstract/Keywords | #1: Research data OR “Research evidence” OR “Research finding*” OR “Survey data” OR “Scientific evidence” OR “Research result*” OR “Scientific data” |
| Title/Abstract/Keywords | #2: Research data sharing OR “Information sharing” OR “Data sharing” OR “Data exchange” OR “Exchange of data” OR “Data transfer” OR “Open data” |
| All fields | #3: Chronic disease* OR “Non communicable disease*” OR “Cancer” OR “Diabetic” OR “Stroke” OR “Arthritis*” OR “Epilepsy” OR “Obesity” OR “Chronic illness” OR “Depression” OR “cardiovascular disease*” OR “Alzheimer’s disease*” OR “chronic kidney disease*” OR “Osteoporosis” OR “Type 2 diabetes” |
| Title/Abstract/Keywords | #4: Barrier* OR “Challenge*” OR “Problem*” OR “Obstacle*” |
| Title/Abstract/Keywords | #5: Elderly OR “Aged” OR “Older” |
| Title/Abstract/Keywords | #6: Low- and middle-income country* OR “Economically developing countries*” OR “Developing countries*” OR “Third World Countries” OR “Developing Economies” |
|  | #1 AND # 2 AND #3 AND # 4 AND # 5 AND # 6 |

**Table 5: Taylor and Francis (TF) = 357**

| Filter | Search statement |
| --- | --- |
| All fields | #1: Research data OR “Research evidence” OR “Research finding*” OR “Survey data” OR “Scientific evidence” OR “Research result*” OR “Scientific data” |
| All fields | #2: Research data sharing OR “Information sharing” OR “Data sharing” OR “Data exchange” OR “Exchange of data” OR “Data transfer” OR “Open data” |
| All fields | #3: Barrier* OR “Challenge*” OR “Problem*” OR “Obstacle*” |
| All fields | #4: Elderly OR “Aged” OR “Older” |
| All fields | #5: Low- and middle-income country* OR “Economically developing countries*” OR “Developing countries*” OR “Third World Countries” OR “Developing Economies” |
|  | #1 AND # 2 AND #3 AND # 4 AND # 5 |

**Table 6: PubMed = 581**

| Filter | Search statement |
| --- | --- |
| All fields | #1: Research data OR “Research evidence” OR “Research finding*” OR “Survey data” OR “Scientific evidence” OR “Research result*” OR “Scientific data” |
| All fields | #2: Research data sharing OR “Information sharing” OR “Data sharing” OR “Data exchange” OR “Exchange of data” OR “Data transfer” OR “Open data” |
| All fields | #3: Barrier* OR “Challenge*” OR “Problem*” OR “Obstacle*” |
| All fields | #4: Elderly OR “Aged” OR “Older” |
| All fields | #5: Low- and middle-income country* OR “Economically developing countries*” OR “Developing countries*” OR “Third World Countries” OR “Developing Economies” |
|  | #1 AND # 2 AND #3 AND # 4 AND # 5 |

**Table 7: Google Scholar (GS) = 150**

| Filter | Search statement |
| --- | --- |
| All fields | #1: Research data OR “Research evidence” OR “Research finding*” OR “Survey data” OR “Scientific evidence” OR “Research result*” OR “Scientific data” |
| All fields | #2: Research data sharing OR “Information sharing” OR “Data sharing” OR “Data exchange” OR “Exchange of data” OR “Data transfer” OR “Open data” |
| All fields | #3: Barrier* OR “Challenge*” OR “Problem*” OR “Obstacle*” |
| All fields | #4: Elderly OR “Aged” OR “Older” |
| Title/Abstract | #5: Low- and middle-income country* OR “Economically developing countries*” OR “Developing countries*” OR “Third World Countries” OR “Developing Economies” |
|  | #1 AND # 2 AND #3 AND # 4 AND # 5 |
